# Influence of Fish Handling Practices Onboard Purse Seiners on Quality Parameters of Sardines (*Sardina pilchardus*) during Cold Storage

**DOI:** 10.3390/biom13020192

**Published:** 2023-01-17

**Authors:** Tibor Janči, Tonka Gauta, Predrag Putnik, Danijel Kanski, Mario Lovrinov

**Affiliations:** 1 Faculty of Food Technology and Biotechnology, University of Zagreb Pierottijeva 6, 10000 Zagreb, Croatia; 2 Marikomerc d.o.o., Grabi 54, 23241 Poličnik, Croatia; 3 Department of Food Technology, University North, Trg dr. Žarka Dolinara 1, 48000 Koprivnica, Croatia; 4 Barbaburaz, obrt za savjetodavne usluge, vl. Danijel Kanski, Kardinala dr. Alojza Stepinca 66, 23211 Pakoštane, Croatia; 5 Maribu d.o.o., Put za Marleru 29, 52204 Ližnjan, Croatia

**Keywords:** sardine, quality, cold storage, lipid oxidation, proteolytic changes

## Abstract

Small pelagic fish are a rich source of high-quality proteins and omega-3 fatty acids, but they are highly perishable due to the activity of microorganisms, endogenous enzymes, and oxidation processes that affect their muscle tissues during storage. This study focused on analyzing the influence of fish handling practices onboard vessels on sensory quality attributes, pH, water holding capacity, TVB-N, proteolytic changes, and lipid oxidation in sardine muscle tissue during cold storage. Experiments were conducted onboard fishing vessels during regular work hours, with added consistency, accounting for similar sardine sizes (physiological and reproductive stages) under similar environmental conditions. Traditional handling practices, e.g., boarding the catch with brail nets and transporting the fish in plastic crates with flake ice, were compared with the use of modified aquaculture pumps for boarding the catch and transporting it in isothermic boxes submerged in ice slurry. Results confirmed significant differences in the parameters among the different fishing vessels, although no significant differences were found between the two methods of fish handling on board the vessels. The study also confirmed a higher rate of lipid oxidation in fish muscle due to physical damage and an increased degree of proteolysis in samples with lower muscle pH values.

## 1. Introduction

The European sardine (*Sardina pilchardus*) is a small pelagic fish common in the Mediterranean Sea, the Adriatic Sea, and the Black Sea, where it is the most marketed and consumed fishery product [1,2]. It is a rich source of high-quality proteins, minerals, and polyunsaturated fatty acids (PUFA), especially n-3 PUFA [2,3,4]. Consumption of n-3 PUFA, especially essential eicosapentaenoic acid (EPA) and docosahexaenoic acid (DHA), plays an important role in a balanced diet and the prevention of various health disorders [5,6,7,8]. Due to its chemical composition, neutral pH, and delicate structure, sardines are also highly perishable and susceptible to rapid quality deterioration due to microbial activity, autolysis, and oxidation processes that develop from the time of death and are influenced by a variety of factors during storage [9,10,11,12].

It is well known that proper storage of fish, i.e., rapid cooling to temperatures near freezing and maintaining the cold chain, is critical for maintaining fish quality [13,14,15]. In addition, there are a variety of different factors that affect the quality of fish meat, such as the cooling method, the time between capture and processing [13,15,16], physical damage caused by the type of fishing gear [17,18], the equipment used to manipulate the catch, e.g., brail nets or pumps [19], or pre-mortem stress that affects biochemical processes and quality parameters in the post-mortem period [20,21,22]. All of these factors have interrelated and irreversible effects on quality parameters that can lead to significant nutritional, health, safety, and economic losses along the fish supply chain.

Continuous advances in fishing equipment and technology result in different fish handling practices on board fishing vessels, which can potentially affect fish quality during storage. Aside from differences in fishing techniques and gear, most differences onboard fishing vessels can be observed in the equipment used to load the catch onboard and in the methods used to cool and maintain the cold chain during transport to the landing site. The use of pumps allows for faster loading of the catch compared with brail nets and minimizes the time fish are crowded within the net, which is important for maintaining the quality of the catch, although several studies have reported higher levels of physical/mechanical damage to the catch with negative effects on quality [19,23]. It is also known that cooling the catch with flake ice or chilled seawater or ice slurry during loading and transport to the landing site affects the quality parameters of different fish species [13,14,15].

It is expected that the live fish pumps used in this study for loading the catch will minimize physical damage to the catch and improve quality due to their design and primary purpose of transporting live fish in aquaculture farms. There are no reports in the available literature on the use of such pumps aboard fishing vessels and their effects on catch quality. Therefore, the objective of this work was to investigate possible differences in quality parameters during the storage of sardines handled by the traditional practice (i.e., loaded with brail nets, cooled in an ice slurry, and transported in flake ice) and the modified practice using modified live fish pumps to minimize physical damage, and direct cooling and transport in isothermic boxes with ice slurry.

## 2. Materials and Methods

### 2.1. Samples

Sardines were sampled during four fishing trips aboard Croatian commercial purse seine vessels in the northern Adriatic Sea (20 nautical mile radius around coordinates 44°09.699′ N, 14°31.619′ E) in October 2020. Of the four vessels that participated in this study (Table 1), catches on board two vessels (A and B) were handled by the traditional practice, i.e., loaded onboard using brail nets and cooled in a mixture of seawater and ice for 20 minutes (300 kg of fish, 150 kg of ice, and 150 L of chilled seawater). After the 20-min cooling period, the fish were separated from the liquid cooling medium and transported in plastic crates with the addition of flake ice (approximately 6 kg of fish to 1 kg of ice). On board vessels C and D, catches were loaded by pumping, using Pescamotion 6 plus aquaculture live fish pumps (Faivre, Baume-les-Dame, France), cooled, and transported in isothermic boxes containing 300 kg of fish, 150 kg of ice, and 150 L of chilled seawater. During fishing operations, sea and air temperatures, the time taken to load the catch on board the vessel, and the total amount of catch were measured.

All sample batches were handled in the same manner: 6 hours after loading onto the vessel, they were transported to the laboratory, visually inspected for physical damage, packed in smaller plastic containers with the addition of flake ice, and stored in the refrigerator at 0 °C until the end of shelf life as determined by sensory evaluation.

Each day, 15 fish from each batch were taken for sensory evaluation of freshness and then gutted, descaled, and filleted. Fillets with skin from each batch were pooled, homogenized with a hand blender, and used for further analyses. All analyses were carried out in triplicate.

### 2.2. Initial Inspection and Chemical Composition Analysis

Upon arrival at the laboratory, the internal temperature of the fish was measured, and one hundred specimens were taken from each batch, visually inspected for physical damage, and their average weight determined. Ten fish from each batch were filleted, pooled, and homogenized with the skin. The homogenized muscle tissue was analyzed for crude chemical composition and fatty acid composition to determine differences with potential influence on quality parameters during cold storage.

Moisture, protein, and ash content were determined according to the methods recommended by the AOAC [24]. Lipid content was determined according to the method of Smedes [25].

Fatty acid methyl esters (FAME) were prepared by transesterification with KOH solution in methanol (c = 2 mol/L) according to the ISO 5509:2000 method [26] and analyzed by an Agilent Technologies 6890N Network GC System gas chromatograph (Santa Clara, CA, USA) with flame ionization detector. FAMEs were separated on a DB-23 capillary column (60 m × 0.25 mm × 0.25 μm, Agilent Technologies, Santa Clara, CA, USA). Helium was used as the carrier gas with a constant flow of 1.5 mL/min. The temperature of the injector was set at 250 °C and that of the detector at 280 °C. The oven temperature was programmed to increase by 7 °C/min from an initial 60 °C to the final temperature of 220 °C, which was maintained for 17 min. The split ratio was 30:1 and the fatty acids were identified by comparing their retention times of 37 Component FAME Mix (Supelco, Sigma-Aldrich, St. Louis, MO, USA). The surface normalization method was used to determine the quantitative composition of the fatty acids, expressed as a percentage of total fatty acids.

### 2.3. Sensory Evaluation

Sensory evaluation of freshness was performed according to the Quality Index Method (QIM) for sardines, as described in the work of Garcia and Careche [15]. Each day, the QIM was performed on 15 samples from each batch by 3 evaluators. The resulting score for each day was calculated as the arithmetic mean of demerit points assigned by all evaluators for the samples inspected, and the end of shelf life was determined when the maximum score of 29 demerit points was reached.

### 2.4. pH, Water-Holding Capacity (WHC), and Total Volatile Basic Nitrogen (TVB-N)

Muscle pH was determined at room temperature according to the method of Vyncke [27]. Ten grams of homogenized muscle tissue was suspended in 100 mL of distilled water and mixed. The pH was determined using a 704 pH Meter with glass electrode 6.0236.100 (Metrohm, Filderstadt, Germany).

The filter paper press method developed by Grau and Hamm [28] was used to measure the amount of water expressed from a minced sample kept under pressure, which is inversely proportional to the WHC. The amount of water expressed (w) was calculated as a percentage of sample weight, according to Equation (1).

(1) w = 100 × (m_2_ − m_1_)/m_S_,

where m_1_ is the weight of the filter paper before compression, m_2_ is the weight of the filter paper after the compression of the sample, and m_S_ is the weight of the sample.

The TVB-N content of the samples was determined by direct distillation as described by Antonacopoulos and Vyncke [29].

### 2.5. Proteolytic Index

The proteolytic index was analyzed according to a modified method of Doi, Shibata, and Matoba [30]. The method is based on the quantification of the total number of amino acids present in the sample (expressed as L-leucine; Leu, Bethesda, MD, USA) after derivatization with Cd-ninhydrin. Two grams of the sample was homogenized in 20 mL of ice-cold 0.01 M HCl using an Ultra Turrax T18 basic homogenizer (IKA Werke GmbH & Co. KG, Baden-Württenberg, Germany) for 60 s on ice and centrifuged for 20 min/7500× *g* at 4 °C in a Rotina 380 R centrifuge (Hettich LabTechnology, Tuttlingen, Germany). A volume of 400 μL of the supernatant was taken and mixed with 800 μL of absolute ethanol and stored at room temperature for 30 min to precipitate the proteins. The solutions were centrifuged at 12,000 rpm for 5 min in a MicroCL 21 microcentrifuge (ThermoFisher Scientific, Waltham, MA, USA). The 400 μL of supernatant was withdrawn and mixed with 800 μL of Cd-ninhydrin reagent, heated in a heating block (Stuart SBH130D, Cole-Parmer Ltd., Stone, UK) for 5 min at 84 °C, cooled on ice (t = 15 min), and centrifuged at 12,000 rpm for 5 min. A calibration curve was plotted for each day of measurement using the Leu solution. The proteolytic index was calculated by measuring the absorbance at 490 nm using Leu as a standard [31]. The results were expressed as mg of total amino acids/g of a sample.

### 2.6. Lipid Oxidation

The degree of lipid oxidation was measured using the 2-thiobarbituric acid reactive substances test (TBARS) as described by Bruna et al. [32] with slight modifications. Five grams of the sample was homogenized in 20 mL of 5% trichloroacetic acid for 3 min in an ice bath. To prevent further oxidation, 0.5 mL of an ethanolic solution of 0.19 M butylated hydroxytoluene (BHT) was added. The homogenate was centrifuged (3000 rpm, 5 min, 5 °C) and filtered through Whatman No. 54 filter paper. An aliquot of 4 mL was mixed with 4 mL of a 0.02 M TBA solution and heated at 100 °C for 30 min. After cooling, the mixture was centrifuged at 3000 *g* for 15 min at 5 °C. Finally, the absorbance was measured at 532 nm. The results were expressed as mg malondialdehyde (MDA)/kg of a sample.

### 2.7. Statistical Analysis

Statistical analysis was performed using the SPSS 17.0 program (StatSoft Inc., Tulsa, OK, USA). Significant differences in observed parameters between batches from different vessels were determined by one-way analysis of variance (ANOVA) followed by posthoc Tukey honest significance (HSD) at the *p* ≤ 0.05 significance level. Student’s *t*-test (*p* ≤ 0.05) was performed to test differences between two examined fish handling practices. The results of all measurements were expressed as mean ± standard deviation.

## 3. Results and Discussion

### 3.1. Parameters of Fishing Operation, Initial Inspection, and Chemical Composition of Fish Samples

The environmental parameters, catch amount, and catch loading time for each vessel are shown in Table 2. It can be noted that the environmental parameters were stable with a constant sea temperature of 19 °C and air temperatures in the range of 16–18 °C, and the catch was low (below 20% of the capacity of each vessel) during all four fishing trips. Although the calculated loading capacity of the modified method using pumps is approximately twice the capacity of the traditional method, the low catch allowed for rapid loading of all four batches and minimized the possibility of quality degradation of all sample batches.

Initial inspection of samples and chemical composition analysis were performed to assess the uniformity of samples from different batches and to identify possible factors that could affect the quality parameters studied during storage.

The results presented in Table 3 show little variation in the core temperature of the samples (0.3–0.9 °C), the similar average weight of the samples, and no significant differences in crude protein content.

The lipid content of the samples ranged from 4.04–7.15 g/100 g and PUFA content ranged from 41.95–46.19%. Statistical analysis showed significant differences between all batches in terms of these parameters, which is important to note as this could affect TBARS results due to the susceptibility of PUFA to oxidation. The results related to chemical composition (Table 3) and fatty acid profile (Table 4) are consistent with previously reported data, without significant differences [1,2].

**Figure 1:**
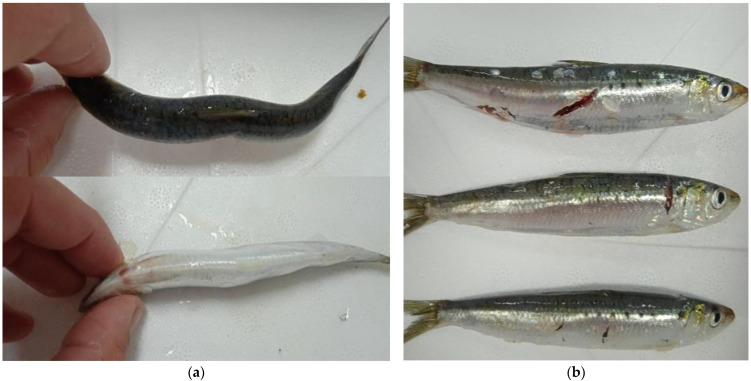
Observed physical damage in samples: (**a**) indentations and irregular shape on samples A and B loaded by brail net and (**b**) damaged skin on sample C loaded by pump.

Visual inspection revealed different types and extents of physical damage to the specimens (Figure 1), i.e., 10–16% of samples A and B handled by the traditional method had visible indentations and irregular body shapes. Because the brail net used to load the catch had a capacity of about 100 kg, the lower layer of the fish was compressed by the weight of the catch above it, which probably caused these kinds of deformities. Sample D, loaded by pumping, had a minimal number of damaged specimens (7%) in the form of light scratches. Sample C, which was also loaded by pumping, had the greatest number of damaged specimens (17%) in the form of deep scratches and cuts on the skin surface. The damage observed was because batch C was a mixed catch containing approximately 20% horse mackerel (*Trachurus* sp.), which damaged the rest of the catch by contact with sharp scales along their lateral lines.

Because the modified method includes transport in a liquid medium (ice slurry), no influence of weight could cause deformity and bruising of the fish, but there is some degree of movement within isothermic boxes during transport due to sea conditions and handling at the landing site. These conditions allow for close contact of the sardines floating in the liquid medium with the sharp scales of the horse mackerel, which is likely the cause of the deep scratches on the samples from batch C. The fact that batches C and D were handled in the same way and the only difference between them was the high proportion of horse mackerel in batch C leads to the conclusion that loading fish with this type of pump is unlikely to result in major physical damage, as has been reported in previous studies with other types of pumps [19].

### 3.2. QIM

**Figure 2:**
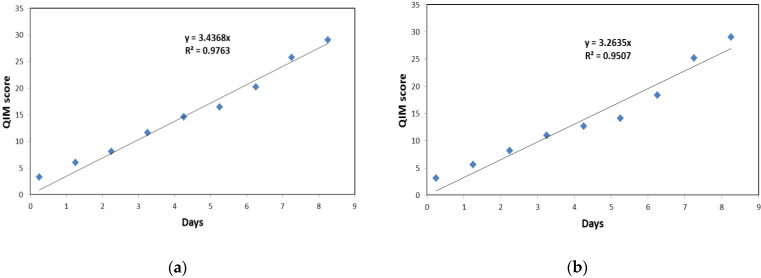
Linear QIM models for (**a**) traditional fish handling practice and (**b**) modified fish handling practice using pumps and isothermic boxes.

Sensory evaluation of freshness was performed to determine the shelf life of the different batches of samples and to observe the dynamics of freshness loss, possibly influenced by the different fish handling practices. The results showed that the samples of all batches were assigned a maximum of 29 demerit points on the eighth day of storage, indicating the end of shelf life. To evaluate the dynamics of freshness loss, linear QIM models were constructed for each sample batch as well as for each handling practice based on the average score of two batches handled by each practice (Figure 2).

The parameters of the obtained models and the theoretical shelf life, calculated as the time required for each sample to reach a maximum of 29 demerit points, are shown in Table 5. The coefficients of determination were in the range of 0.9401–0.9763, indicating the high reliability of all obtained models. Slight variations in theoretical shelf life were observed between all batches, and the results showed about 12 hours longer shelf life of samples handled by modified compared with that of traditional practice, although these differences had no statistical significance but questionable practical significance. Overall, the shelf life of sardines in this work is consistent with previously reported data [15].

### 3.3. pH, Water-Holding Capacity (WHC), and Total Volatile Basic Nitrogen (TVB-N)

**Figure 3:**
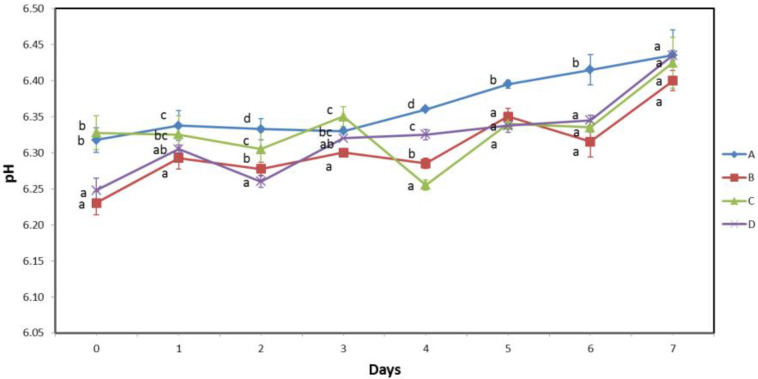

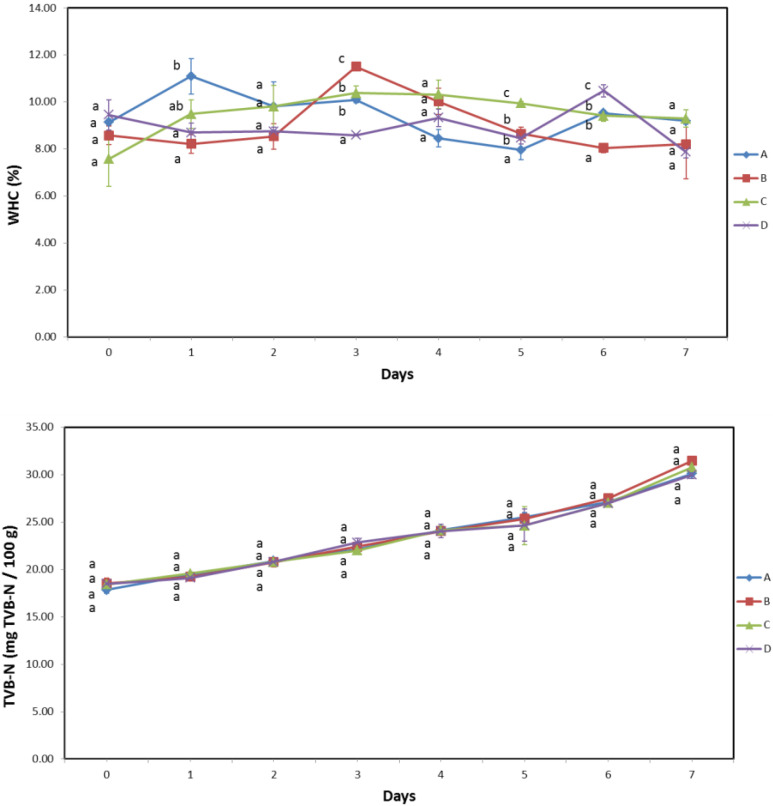
pH value of muscle tissue, water holding capacity, and TVB-N content of samples A and B handled by the traditional method, and samples C and D handled by modified handling method during 7 days of storage at 0 °C. Capital letters A, B, C and D (legend) indicate different sample batches. Lowercase letters (a, b, c, d) on the same day indicate significant differences among different sample batches (*p* ≤ 0.05).

Measurement of muscle pH revealed initial pH values ranging from 6.23 to 6.33, whereas statistical analysis revealed a significantly lower pH for samples B and D compared with samples A and C (Figure 3). This relatively narrow pH interval suggests that there was no significant difference in pre-mortem stress between batches of fish loaded onto vessels with brail nets or pumps, as extensive pre-mortem stress leads to an accumulation of lactate and a significant drop in muscle pH [20,22].

The depletion of energy reserves and the drop in muscle pH have significant effects on postmortem changes in fish muscle, such as the early onset of rigor mortis [22,33,34] and various quality parameters such as muscle color [35], autolysis rate [19,33,36], and water holding capacity [20,22]. The initial muscle pH values are consistent with previously reported data for sardines [37,38,39] and indicate a similar level of pre-mortem stress, which is to be expected because all studied vessels caught relatively small amounts at this time (below 20% of maximum vessel capacity) and the total loading time of all batches was less than 30 min.

The changes in pH during storage show small, although statistically significant, variations between the different batches, following no clear trend. In all cases studied, pH values increased with storage time, which is in agreement with previous studies [37,38,39]. Statistical analysis revealed no significant differences between the two handling practices during the entire shelf life.

Water holding capacity measurements (Figure 3) showed no significant differences on the first day of the experiment and slight variations throughout the shelf life. Although in some cases the differences in WHC values were statistically significant, no clear trend is evident. These results are likely because pre-mortem stress and muscle pH levels were similar, as discussed above, and storage conditions were the same throughout the shelf life. In addition, no significant differences were observed between the two treatment methods throughout the experiment.

TVB-N content increased during storage from initial values between 17.85 and 18.51 mg TVB-N/100 g to 29.97–31.45 mg TVB-N/100 g on the last day of the experiment (Figure 3). This was consistent with previously reported data [15,37,40]. Data analysis did not reveal any significant differences between the different sample batches or between handling practices at any time during the experiment, indicating that the growth and activity of the organisms responsible for the formation of TVB-N compounds were not affected by the handling practices and the initial differences between the sample batches, but only by the storage time.

### 3.4. Proteolytic Index

**Figure 4:**
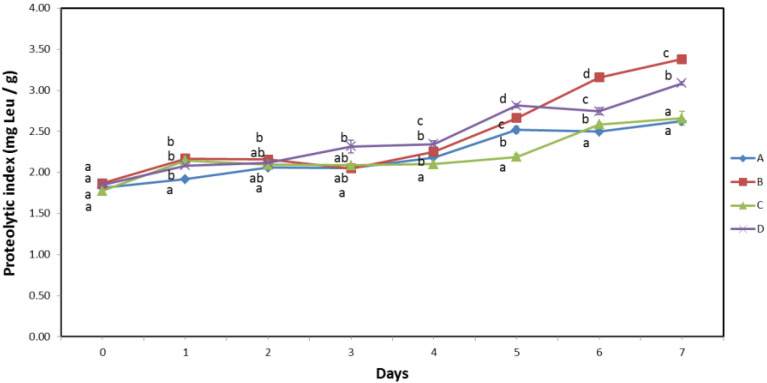
Proteolytic index of samples A and B handled by the traditional method, and samples C and D handled by the modified handling method during 7 days of storage at 0 °C. Capital letters A, B, C and D (legend) indicate different sample batches. Lowercase letters (a, b, c, d) on the same day indicate significant differences among different sample batches (*p ≤* 0.05).

The determination of the degree of proteolysis showed no significant difference between the different batches on the first day of analysis and the values ranged from 1.78–1.87 mg Leu/g, as shown in Figure 4. This was expected due to the short time from capture to analysis and the fact that proteolysis occurs post-mortem due to the activity of endogenous enzymes [20,33,36,41] and proteolytic microorganisms [14], which progresses at a certain rate from the time of death to the end of shelf life. As proteolysis progressed during the storage of the samples, slight but statistically significant differences were observed between batches from the second to the fourth day of storage. From the fifth day of storage until the end of shelf life, a clear separation of samples B and D with higher values of proteolysis index from samples A and C with lower values is observed.

Since samples A and C were handled by different practices, as were samples B and D, statistical analysis in this experiment revealed no significant differences between the two handling practices in this experiment. However, samples A and C had significantly higher pH than samples B and D on the first day of the experiment (Figure 3). Data analysis revealed clear and significant differences (*p* ≤ 0.05) between samples grouped by initial pH from the fifth day of storage. This can be attributed to an increased autolysis rate due to a lower initial muscle pH [19,33,36,41], as described in detail in the previous sections. Because the difference in initial muscle pH was minimal, the effect of promoted autolysis was not clearly observed during the first four days, but only during the later stages of storage when autolysis products accumulated in muscle tissue.

### 3.5. Lipid Oxidation

**Figure 5:**
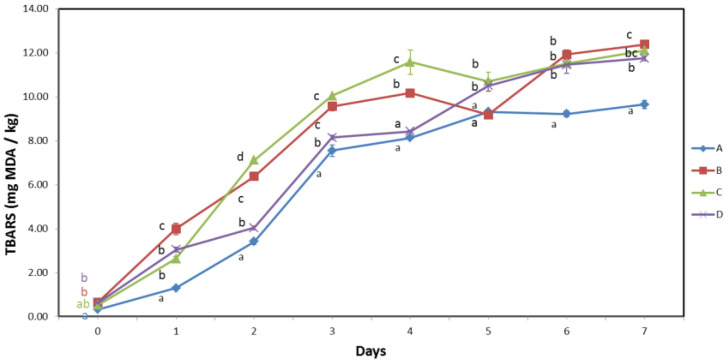
TBARS of samples A and B handled by the traditional method, and samples C and D handled by the modified handling method during 7 days of storage at 0 °C. Capital letters A, B, C and D (legend) indicate different sample batches. Lowercase letters (a, b, c, d) on the same day indicate significant differences among different sample batches (*p* ≤ 0.05).

As expected for samples with high PUFA content, TBARS values of all batches increased significantly during storage [42,43,44], as shown in Figure 5. The initial TBARS values ranged from 0.33 to 0.64 mg MDA/kg, with sample A having a significantly lower TBARS value compared with those of samples B and D, while no significant difference was observed for sample C compared with those of either sample A or samples B and D. The TBARS values of all batches significantly increased during the storage period. Throughout the storage period, except for days 4 and 5, sample A had significantly lower TBARS values compared with those of all other samples, and at the end of storage, the TBARS value increased to 9.65 mg MDA/kg. The other batches followed the same trend, although the statistical significance of the differences between batches B, C, and D was not as consistent. On the last day, the TBARS value of batches B, C, and D varied between 11.76 and 12.39 mg MDA/kg, which is consistent with other studies [2,13,15].

Because the negative effects of lipid oxidation products on the flavor of fresh sardines are not a major concern due to the strong off-flavors produced by microbial activity at the end of the shelf life, the true value of the results obtained is reflected in the fact that lipid oxidation affects the most nutritionally valuable polyunsaturated fatty acids. Although the results obtained cannot be used for quantification purposes, the low TBARS values in sample A indicated the highest degree of PUFA preservation and the lowest loss of nutritional value among all the samples studied.

Statistical analysis showed a significant difference between the two handling methods only on the fifth day of storage, due to the relatively low TBARS value of sample B. Nevertheless, sample B had the highest TBARS value on the last two days of storage, therefore no conclusions can be drawn about differences between the two handling practices.

It is interesting to observe the difference in the rate of lipid oxidation in sample C compared with that of the other samples. The initial measurement showed that sample C had the second lowest TBARS value, which rapidly increased over the next four days. Sample C had the highest TBARS value on days 2, 3, and 4, which was significantly different from that of all other samples. Although sample C had the lowest total PUFA content (Table 3), calculated as % crude fat × % PUFA, lipid oxidation occurred at a significantly higher rate than in the other samples. This can be explained by the specific type of physical damage observed in sample C, as previously described (Figure 1). Sample C, unlike the other samples, had open scratches and cuts on the skin surface, exposing the muscle tissue to oxygen, which significantly increased the lipid oxidation rate. This conclusion is supported by the fact that the damage mainly occurred in the lateral region of the fish, where the dark muscle is located just below the skin surface. It is well known that fish dark muscle is highly susceptible to lipid oxidation because it naturally contains greater amounts of lipids [45,46] as well as myoglobin, which acts as a prooxidant in these reactions [47,48,49]. This is consistent with previous studies on herring, which concluded that the presence of prooxidants and the influence of oxygen contact and interactions with tissue affect the course of lipid oxidation more than the lipid composition of the sample [50].

## 4. Conclusions

The results showed significant differences in sardine quality parameters between the different fishing vessels and no clear and significant differences were found between the fish handling practices studied, which can be attributed to the moderate environmental conditions and low catches, which allowed rapid loading and cooling of the catch by both handling practices. 

Nevertheless, the calculated loading capacity of the modified method with pumps is twice that of the traditional method with brail nets, suggesting that the use of pumps may be beneficial in warm conditions and large catches by minimizing the time the fish spends at high temperatures before cooling. In addition, physical damage to the catch in this study cannot be attributed to the use of this type of pump. Single-stage cooling and transport in isothermic boxes with a liquid medium minimized the possibility of compression and deformation of the catch, although this was found to promote scratches and skin damage during transport in the case of mixed species in the catch. 

This study also showed that a significantly higher rate of proteolytic changes during storage was observed in samples with lower initial muscle pH values and a higher rate of lipid oxidation was observed in samples with mechanically damaged skin. Further studies are warranted to investigate the differences in the stability of sardine biomolecules under different handling practices, taking into account the relevant parameters identified in the current work.

## Figures and Tables

**Table 1:** Overview of handling practices and operations onboard vessels A, B, C, and D.

Sample	Handling Practice	Loading Method	Cooling Method	Transport
**A**	Traditional	Brail net	Mixture of chilled seawater and ice	Plastic crates, with addition of flaked ice
**B**				
**C**	Modified	Live fish pump	Mixture of chilled seawater and ice	Isothermic box, in mixture of chilled seawater and ice
**D**				

**Table 2:** Sea and air temperature, total amount of catch, loading time, and loading capacity during collection of samples A and B were handled by the traditional method, and samples C and D were handled by the modified handling method.

Sample	Sea Temperature (°C)	Air Temperature (°C)	Total Amount of Catch (kg)	Loading Time (min)	Loading Capacity (kg/min)
**A**	19	16	2630	27	97.4
**B**	19	16	1170	14	83.6
**C**	19	16	3601	19	189.5
**D**	19	18	1820	11	165.5

**Table 3:** Temperature, average weight, number of samples with visible physical damage, crude protein and fat content, and saturated (SFA), monounsaturated (MUFA), and polyunsaturated (PUFA) fatty acid content of samples A and B handled by traditional method, and samples C and D handled by modified handling method.

Sample	Temperature (°C)	Average Weight (g)	Damaged Samples	Protein g/100 g	Fat g/100 g	SFA % t.f.a.	MUFA % t.f.a.	PUFA % t.f.a.
**A**	0.5	22.1 ± 1.9 ^a^	16/100	20.87 ± 0.02 ^a^	6.03 ± 0.02 ^c^	36.99 ± 0.19 ^b^	18.64 ± 0.25 ^c^	43.57 ± 0.02 ^b^
**B**	0.9	24.8 ± 2.3 ^a^	10/100	21.10 ± 0.12 ^a^	5.20 ± 0.23 ^b^	37.54 ± 0.02 ^bc^	17.20 ± 0.02 ^b^	43.98 ± 0.01 ^c^
**C**	0.3	23.1 ± 1.8 ^a^	17/100	21.26 ± 0.25 ^a^	4.04 ± 0.30 ^a^	36.37 ± 0.15 ^a^	16.16 ± 0.00 ^a^	46.19 ± 0.12 ^d^
**D**	0.7	24.0 ± 2.1 ^a^	6/100	20.67 ± 0.27 ^a^	7.15 ± 0.05 ^d^	37.41 ± 0.12 ^c^	19.40 ± 0.07 ^d^	41.95 ± 0.10 ^a^

Different letters (a, b, c, d) in the same column indicate significant differences among different sample batches (*p* ≤ 0.05). Fatty acids content is expressed as % of total fatty acids (% t.f.a.).

**Table 4:** Fatty acid composition of samples A and B were handled by the traditional method, and samples C and D were handled by the modified handling method. Results expressed as % of total fatty acids.

Fatty Acid	Sample A	Sample B	Sample C	Sample D
C12:0	0.25 ± 0.00 ^b^	0.25 ± 0.00 ^b^	0.13 ± 0.00 ^a^	0.25 ± 0.00 ^b^
C14:0	8.01 ± 0.06 ^d^	7.19 ± 0.01 ^b^	5.62 ± 0.01 ^a^	7.75 ± 0.04 ^c^
C14:1	0.04 ± 0.00 ^a^	0.03 ± 0.00 ^a^	0.25 ± 0.01 ^b^	0.04 ± 0.00 ^a^
C15:0	0.88 ± 0.01 ^a^	0.94 ± 0.00 ^b^	1.03 ± 0.01 ^c^	0.87 ± 0.00 ^a^
C16:0	20.66 ± 0.09 ^a^	21.42 ± 0.02 ^b^	21.80 ± 0.04 ^c^	21.27 ± 0.08 ^b^
C16:1	6.38 ± 0.04 ^d^	5.18 ± 0.02 ^b^	3.09 ± 0.01 ^a^	6.06 ± 0.04 ^c^
C17:0	1.00 ± 0.00 ^b^	1.11 ± 0.00 ^c^	1.21 ± 0.00 ^d^	0.97 ± 0.00 ^a^
C17:1	0.10 ± 0.00 ^a^	0.10 ± 0.01 ^a^	0.08 ± 0.03 ^a^	0.10 ± 0.00 ^a^
C18:0	5.01 ± 0.02 ^a^	5.63 ± 0.02 ^d^	5.48 ± 0.02 ^c^	5.18 ± 0.00 ^b^
C18:1c	10.72 ± 0.30 ^a^	10.62 ± 0.01 ^a^	11.25 ± 0.02 ^b^	11.67 ± 0.03 ^b^
C18:2c	2.00 ± 0.01 ^a^	2.14 ± 0.10 ^a^	2.14 ± 0.00 ^a^	2.04 ± 0.01 ^a^
C18:3n6	0.18 ± 0.00 ^c^	0.15 ± 0.01 ^b^	0.10 ± 0.00 ^a^	0.17 ± 0.01 ^bc^
C18:3n3	1.22 ± 0.01 ^b^	1.09 ± 0.00 ^a^	1.16 ± 0.04 ^ab^	1.30 ± 0.01 ^c^
C20:0	0.91 ± 0.01 ^d^	0.70 ± 0.00 ^b^	0.58 ± 0.00 ^a^	0.86 ± 0.00 ^c^
C20:1	1.22 ± 0.01 ^b^	1.08 ± 0.00 ^a^	1.29 ± 0.02 ^c^	1.33 ± 0.00 ^c^
C20:2	0.42 ± 0.00 ^a^	0.43 ± 0.00 ^a^	0.47 ± 0.00 ^c^	0.45 ± 0.00 ^b^
C20:4n6	1.26 ± 0.01 ^ab^	1.45 ± 0.00 ^c^	1.33 ± 0.04 ^b^	1.18 ± 0.02 ^a^
C20:3n3	0.21 ± 0.00 ^ab^	0.21 ± 0.00 ^a^	0.22 ± 0.00 ^b^	0.23 ± 0.00 ^c^
C20:5n3	11.69 ± 0.03 ^d^	10.94 ± 0.02 ^b^	8.53 ± 0.00 ^a^	11.08 ± 0.02 ^c^
C22:1	0.19 ± 0.00 ^a^	0.19 ± 0.00 ^a^	0.21 ± 0.00 ^b^	0.19 ± 0.00 ^a^
C23:0	0.11 ± 0.00 ^a^	0.11 ± 0.01 ^a^	0.33 ± 0.19 ^a^	0.08 ± 0.00 ^a^
C24:0	0.16 ± 0.00 ^a^	0.20 ± 0.00 ^d^	0.18 ± 0.00 ^c^	0.17 ± 0.00 ^b^
C22:6n3	26.59 ± 0.01 ^b^	27.99 ± 0.07 ^c^	32.72 ± 0.04 ^d^	25.94 ± 0.09 ^a^
* n.i.	0.74 ± 0.02 ^a^	0.85 ± 0.05 ^a^	0.81 ± 0.04 ^a^	0.79 ± 0.09 ^a^

* n.i.—not identified; Different letters (a, b, c, d) in the same row indicate significant differences among different sample batches (*p* ≤ 0.05). Fatty acids content is expressed as % of total fatty acids (% t.f.a.).

**Table 5:** Slope (a), coefficient of determination (R^2^), and theoretical shelf life for linear QIM models for samples A and B handled by traditional method, and samples C and D handled by modified handling method, and for two different fish handling practices (traditional, modified).

Sample	a	R^2^	Theoretical Shelf Life (Days)
**A**	3.3950	0.9715	8.54
**B**	3.4787	0.9778	8.33
**C**	3.2945	0.9401	8.80
**D**	3.2326	0.9523	8.97
**Traditional**	3.4368	0.9763	8.43
**Modified**	3.2635	0.9507	8.89

## Data Availability

Not applicable.
